# Burden and Risk Factors for Coinfections in Patients with a Viral Respiratory Tract Infection

**DOI:** 10.3390/pathogens13110993

**Published:** 2024-11-13

**Authors:** Pierachille Santus, Fiammetta Danzo, Juan Camilo Signorello, Alberto Rizzo, Andrea Gori, Spinello Antinori, Maria Rita Gismondo, Anna Maria Brambilla, Marco Contoli, Giuliano Rizzardini, Dejan Radovanovic

**Affiliations:** 1 Division of Respiratory Diseases, Department of Biomedical and Clinical Sciences (DIBIC), Università Degli Studi di Milano, Ospedale L. Sacco, ASST Fatebenefratelli-Sacco, 20147 Milano, Italy; fiammetta.danzo@unimi.it (F.D.); juan.signorello@unimi.it (J.C.S.); dejan.radovanovic@unimi.it (D.R.); 2 Coordinated Research Center on Respiratory Failure, University of Milan, 20122 Milano, Italy; 3 Laboratory of Clinical Microbiology, Virology and Bioemergencies-ASST Fatebenefratelli-Sacco, Luigi Sacco University Hospital, 20157 Milano, Italy; rizzo.alberto@asst-fbf-sacco.it; 4 Department of Infectious Diseases, L. Sacco University Hospital, ASST Fatebenefratelli-Sacco, 20122 Milano, Italy; andrea.gori@unimi.it; 5 Centre for Multidisciplinary Research in Health Science (MACH), Università Degli Studi di Milano, 20122 Milano, Italy; 6 III Division of Infectious Diseases, ASST Fatebenefratelli-Sacco, Department of Biomedical and Clinical Sciences, Università Degli Studi di Milano, 20157 Milano, Italy; spinello.antinori@unimi.it; 7 Clinical Microbiology, Virology and Bioemergency Diagnostics, ASST Fatebenefratelli-Sacco, Luigi Sacco University Hospital, 20157 Milano, Italy; mariarita.gismondo@unimi.it; 8 Emergency Medicine Unit, Luigi Sacco University Hospital, ASST Fatebenefratelli-Sacco, 20157 Milano, Italy; brambilla.annamaria@asst-fbf-sacco.it; 9 Respiratory Section, Department of Translational Medicine, University of Ferrara, 44121 Ferrara, Italy; ctm@unife.it; 10 I Division of Infectious Diseases, Luigi Sacco University Hospital, ASST Fatebenefratelli-Sacco, 20157 Milano, Italy; rizzardini.giuliano@gmail.com

**Keywords:** influenza, respiratory syncytial virus, SARS-CoV-2, multidrug resistant, coinfection, mortality

## Abstract

Which patients should be monitored for coinfections or should receive empirical antibiotic treatment, in patients with an acute viral respiratory infection, is largely unknown. We evaluated the prevalence, characteristics, outcomes of coinfected patients, and risk factors associated with a coinfection among patients with an acute viral infection. A retrospective, single-center study recruited consecutive patients from October 2022 to March 2023 presenting to the emergency department with signs of a respiratory tract infection. Patients were screened for respiratory viruses and bacterial/fungal secondary infections according to local standard procedures. Outcomes included severe disease, in-hospital complications, all-cause in-hospital and ICU-related mortality, time to death, time to discharge, and time to coinfection. The analysis included 652 patients. A viral infection and a secondary bacterial/fungal infection were detected in 39.1% and 40% of cases. Compared with the rest of the cohort, coinfected patients had more frequently severe disease (88.3%, *p* < 0.001; 51% in patients with SARS-CoV-2) and higher in-hospital mortality (16.5%, *p* = 0.010). Nephropathy (OR 3.649, *p* = 0.007), absence of COVID-19 vaccination (OR 0.160, *p* < 0.001), SARS-CoV-2 infection (OR 2.390, *p* = 0.017), and lower blood pressure at admission (OR 0.980, *p* = 0.007) were independent risk factors for coinfection. Multidrug-resistant pathogens were detected in 30.8% of all coinfections. Patients with a viral infection are at high risk of bacterial coinfections, which carry a significant morbidity and mortality burden.

## 1. Introduction

Acute respiratory tract infections account for significant morbidity in the general population and excessive mortality, particularly in elderly frail patients with concomitant chronic conditions [1,2,3,4]. In the last decades, the development of molecular techniques currently used in daily clinical practice has led to the recognition of viral infections as the main cause of acute respiratory infections. The development of efficient vaccines for the adult population pushed the research to better characterize the clinical burden and risk factors for *Influenza* and *Respiratory Syncytial virus* (RSV) infection. In a global point prevalence real-world study in patients hospitalized with community-acquired pneumonia (CAP) [5], *Influenza A* was detected in almost 23% of tested patients [6]. More recently, we found that in a large population of patients presenting to the emergency department with flu-like symptoms, 8.5% of patients had RSV infection [7], which, interestingly, was associated with a higher incidence of severe disease compared with *Influenza* [7]. RSV infection has also been associated with 8.7% of outpatient-managed Chronic Obstructive Pulmonary Disease (COPD) exacerbations [8]. Viral infections predispose to bacterial super-infection [9,10] by damaging airway epithelium and dysregulating both innate and acquired immune responses [11]. Only a few studies evaluated simultaneously the presence of bacterial and viral infections, and risk factors associated with a secondary infection were mostly explored in patients with CAP, with a particular focus on severe CAP and Intensive Care Unit (ICU) outcomes. Available studies showed that a bacterial coinfection complicating a viral respiratory tract infection is consistently associated with severe disease manifestations, high ICU admission, and high mortality rates [12,13,14,15,16]. However, which patients should be monitored for coinfections or should receive empirical antibiotic treatment is largely unknown. To satisfy the pressing clinical need to identify patients at risk of empirical antibiotic treatment and also the necessity to set an appropriate antibiotic coverage for multidrug-resistant (MDR) bacteria, considering the significant healthcare burden and heterogeneity in viral respiratory infections, the broadening of the knowledge in terms of prevalence, characteristics, and risk factors for bacterial/fungal coinfections represents a compelling clinical needed.

The aim of the present study was to describe the prevalence, clinical characteristics, and outcomes of patients with coinfections among patients accessing the emergency department with an acute viral respiratory infection.

## 2. Materials and Methods

### 2.1. Study Design and Data Collection

This was a retrospective, single-center observational study. The methods and patient selection have been partially described elsewhere [7]. Briefly, we recruited patients referring to the emergency department of the Luigi Sacco University Hospital in Milan (Italy), a secondary care teaching hospital covering a catchment area of 600,000 people, from 1 October 2022 to 31 March 2023, presenting to the emergency department with flu-like or respiratory tract infection symptoms. Patients with respiratory symptoms including Influenza-like illness, signs of upper or lower respiratory tract infection with or without de novo or acute on chronic respiratory failure were screened per protocol for SARS-CoV-2, *Influenza virus* A/B, RSV with a multiplex nasal–pharyngeal swab, and consecutively enrolled as described elsewhere [7]. A multiple molecular panel to screen for additional respiratory viruses was performed based on clinical judgment. Bacterial or fungal infections were evaluated according to local standard procedures in blood, sputum, bronchial aspirate, bronchoalveolar lavage, pleural fluid, fecal, and urine as per clinical indication [17]. Antibiograms were reviewed to identify MDR pathogens following the European Committee on Antimicrobial Susceptibility criteria (EUCAST [18,19]. Testing included carbapenemase-producing *Klebsiella pneumoniae* (KPC), methicillin-resistant *Staphylococcus aureus* (MRSA), *Acinetobacter baumanii*, extended-spectrum beta-lactamase-positive (ESBL) bacteria, vancomycin-resistant Enterococci (VRE), and carbapenem-resistant organisms, including, but not limited to, carbapenem-resistant Enterobacterales and carbapenem-resistant *Pseudomonas aeruginosa*.

The exclusion criteria included (i) <18 years old; (ii) a viral nasopharyngeal swab not performed for acute symptoms (e.g., screening purposes); (iii) skin swabs for in-hospital screening; and (iv) cultural isolates accounted as contaminants by the microbiology laboratory.

Patients that had >1 emergency department access were considered as separate cases only if the two episodes were >30 days apart. The Charlson Comorbidity index [20] was computed for all patients. The type of respiratory support registered included standard oxygen, high flow nasal cannula (HFNC), continuous positive airway pressure (CPAP), non-invasive ventilation (NIV), and invasive mechanical ventilation (IMV). Clinical outcomes included in-hospital complications (occurrence of sepsis, shock, tracheostomy), ICU admission, and in-hospital and ICU-related mortality. A detailed list of variables extracted from electronic charts is reported in the Online data supplement.

### 2.2. Study Objectives

The aims of this study were as follows: (i) to evaluate the prevalence of coinfection in patients tested for viral respiratory infection among patients accessing the emergency department for acute respiratory symptoms; (ii) to compare the clinical characteristics and outcomes of patients with a single viral infection and patients with a secondary bacterial/fungal infection, (iii) to assess risk factors associated with a coinfection; and (iv) to explore the prevalence, characteristics, and outcomes of MDR coinfections.

### 2.3. Study Definitions

Influenza-like illness was defined according to the World Health Organization definition [21] and included signs of a respiratory infection associated with fever, cough, and an onset of <10 days. Immune depression criteria were from Di Pasquale et al. [22]. Chest X-rays or computerized tomography performed within 48 h from admission were singularly reviewed for the presence of interstitial or lobar lung infiltrates and pleural effusion. Bacterial infection or coinfection was considered a positive sputum, bronchial-alveolar lavage, blood culture, pleural liquid culture, or urine culture that occurred at any time from emergency department admission. Additional study definitions are reported in the Online data supplement.

### 2.4. Statistical Analysis

Qualitative and quantitative variables were described as frequencies, mean (standard deviation—DS), or median (interquartile range—IQR) depending on their parametric distribution. Normality was assessed with the Kolmogorov–Smirnov test. Chi-squared and Fisher exact tests were used to compare categorical variables; continuous variables were compared with the Student t-test, Mann–Whitney U, or Kruskal–Wallis tests depending on their distribution, as appropriate.

Logistic regression analysis preceded by the assessment of collinearity was performed to assess the risk of having a coinfection in patients with a positive viral swab compared with the rest of the population. Kaplan Meier survival curves with the Wilcoxon–Breslow–Gehan test were assessed to test the time to coinfection and time to blood and respiratory tract coinfection, length of hospital stay, and in-hospital survival in patients with and without a positive viral swab and time to coinfection and time to blood and respiratory tract coinfection by type of viral infection. Due to the limited number of fungal infections, the latter were not analyzed separately. A two-tailed *p*-value < 0.05 was considered statistically significant. Statistical analyses were performed with IBM SPSS, Statistics for Windows version 21.0 (IBM Corp, Armonk, NY, USA).

## 3. Results

**Figure 1:**
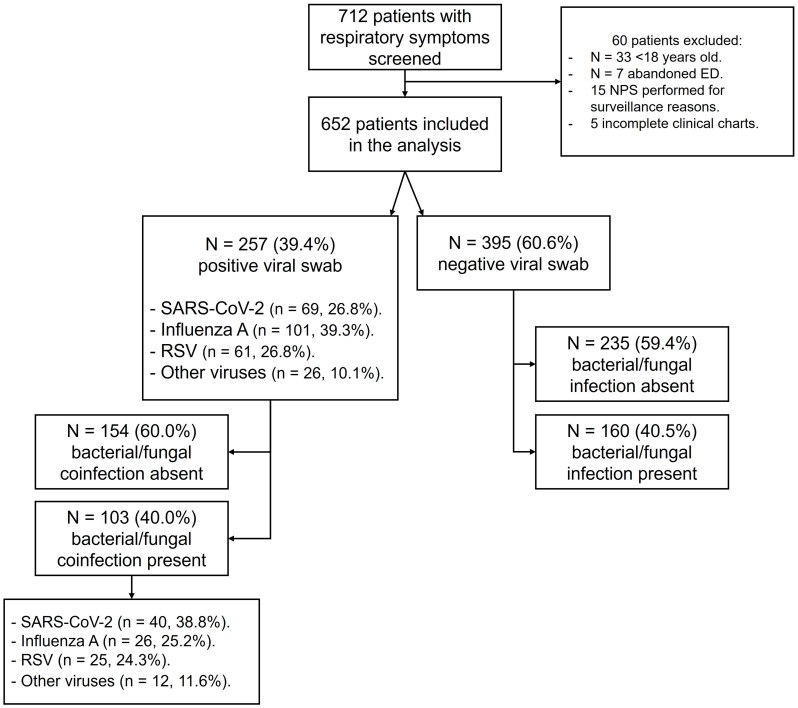
Flow chart describing study groups and prevalence of bacterial/fungal coinfections in patients with and without a positive viral swab.

In total, 712 patients were screened for inclusion criteria, and 652 patients (51.1% males, median (IQR) age 75 (60–84) years) were included in the analysis. The reasons for excluding patients are reported in Figure 1.

### 3.1. Prevalence and Type of Coinfection

**Figure 2:**
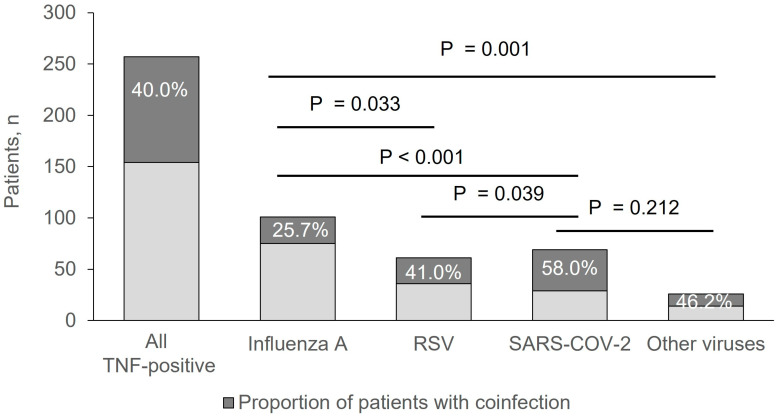
Proportion of coinfected patients (dark grey) and not coinfected patients (light grey) within each virus group.

A positive viral swab was detected in 257/652 patients (39.1%). Bacterial coinfections were found in 103 (40.0%) patients with a positive viral swab. Among patients with a negative viral swab (*n* = 395/652, 60.9%), bacterial or fungal infections were found in 160 (40.5%) patients (*p* = 0.935 vs. positive viral swab group). Within patients with a positive viral swab, 101 (39.3%) had *Influenza A*, 61 (23.7%) had RSV, 69 (26.8%) had SARS-CoV-2, and 26 (10.1%) had other viruses including *Human metapneumovirus*, *Influenza virus B*, *Adenovirus*, *Human Parainfluenza virus*, and *Bocavirus* (Figure 1). The highest prevalence of coinfections was detected in patients with SARS-CoV-2 (58.0%; *p* = 0.039 vs. RSV group and *p* < 0.001 vs. *Influenza A* group), followed by patients with other viruses (46.2%), RSV (41.0%, *p* = 0.033 vs. Influenza A group), and Influenza A (25.7%) (Figure 2).

In the whole sample, the most prevalent pathogens isolated were *Escherichia coli* (9.4%), *Enterobacteriaceae* (5.4%), *Staphylococci* spp. (5.2%), and *Candida* spp. (4.1%) (Supplementary Figure S1). The pathogen distribution did not differ in patients with and without a viral infection (*p* = 0.724 for between-group difference; Supplementary Table S1 and Supplementary Figure S2).

The prevalence of bacterial species was generally independent of the type of viral infection (15.4%; *p* < 0.001 for group comparison) (Supplementary Table S2).

### 3.2. Time-Dependent Outcomes in Patients with Coinfection

**Figure 3:**
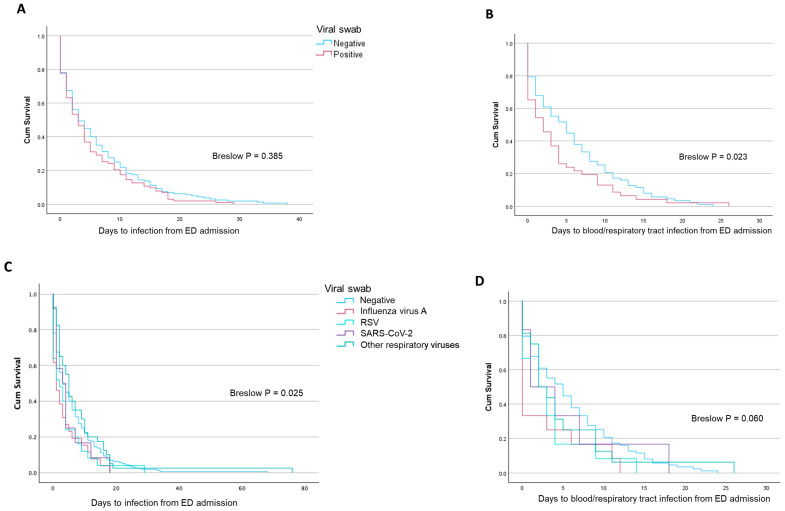
Survival curves reporting the time to infection/coinfection (**A**) and a blood/respiratory tract infection/coinfection (**B**) in patients with and without a positive viral swab. The same is reported for single viral isolates (**C**,**D**).

Time to coinfection was not different in patients with and without a positive viral swab (*p* = 0.385) (Figure 3A), while patients with a positive viral swab developed a blood or respiratory bacterial coinfection more rapidly (*p* = 0.023) (Figure 3B). With respect to patients with a negative viral swab or a viral swab positive for *Influenza A*, time to coinfection from emergency department admission was significantly lower in patients with SARS-CoV-2, RSV, or other viruses (*p* = 0.025) (Figure 3C), while time to blood/respiratory coinfection was independent of the type of viral infection (*p* = 0.060) (Figure 3D).

### 3.3. Clinical Characteristics and Outcomes in Coinfected Patients

**Figure 4:**
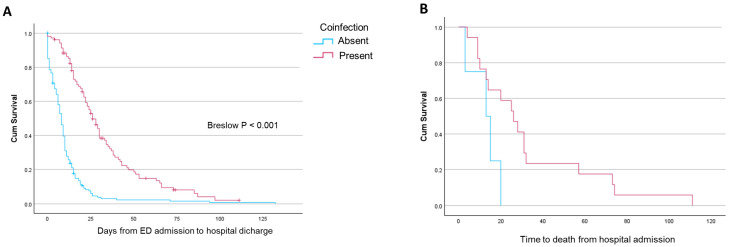
Survival curves reporting the time to hospital discharge (**A**) and time to death from ED admission (**B**) in patients with a positive viral swab and with or without a coinfection.

Clinical and anthropometric characteristics of the study population are reported in Table 1. Compared with the rest of the population, coinfected patients were significantly older, were less frequently covered with COVID-19 vaccination, and had a higher prevalence of chronic heart failure, atrial fibrillation, nephropathy, immune depression, and AIDS (Table 1). Coinfected patients were more likely to present with lung infiltrates, higher inflammatory biomarkers, and a higher chance of having shock or sepsis compared with patients without a coinfection (Table 2). Coinfected patients were significantly more exposed to antibiotics (87.1% vs. 67.5%, *p* < 0.001). Acute respiratory failure (aRF) tended to be more frequent in coinfected patients, that were significantly more exposed to oxygen therapy (82.5%, *p* < 0.001), HFNC (16.5%, *p* = 0.003), CPAP (16.5%, *p* = 0.001), and IMV (16.5%, *p* < 0.001) (Table 2). Coinfected patients had the highest prevalence of severe disease (88.3%, *p* < 0.001) and were at higher risk of being admitted to the ICU and being tracheostomized (20.4% and 10.8%, respectively) (Table 2). Hospital stay was significantly longer in patients with coinfection compared with other groups (25 (14–37) vs. 8 (3–13) and 10 (5–18) days in patients with viral infection alone and the rest of the cohort, respectively, both *p* < 0.001; Table 2 and Figure 4A). Time to in-hospital death from admission tended to be shorter in patients with coinfection (Figure 4B), but in-hospital and ICU-related mortality was significantly higher in coinfected patients (16.5%, *p* = 0.010 and 16.5%, *p* = 0.010 vs. the rest of the cohort) (Table 2).

### 3.4. Risk Factors for Coinfection

Risk factors for having a coinfection were studied in a three-step model, that progressively included clinical, diagnostic, and biochemistry elements to help physician decision-making in the emergency setting. In a model that included heart failure, nephropathy, COVID-19 vaccination, age, bilateral pneumonia, SARS-CoV-2 infection, and CRP, systolic blood pressure and white blood cell count as continuous variables, nephropathy (OR 95%CI: 3.649, 1.422–9.367; *p* = 0.007), not being vaccinated for COVID-19 (OR 95%CI: 0.160, 0.055–0.471; *p* < 0.001), SARS-CoV-2 infection (OR 95%CI: 2.390, 1.165–4.902; *p* = 0.017), and lower blood pressure at admission (OR 95%CI: 0.980, 0.967–0.995; *p* = 0.007) were independent risk factors for coinfection (Models A, B, and C in Table 3). The same was confirmed when age, blood pressure, and white blood cell count were considered as dichotomous variables (Model D in Table 3).

### 3.5. Role of Coinfection in Different Viral Infections

**Figure 5:**
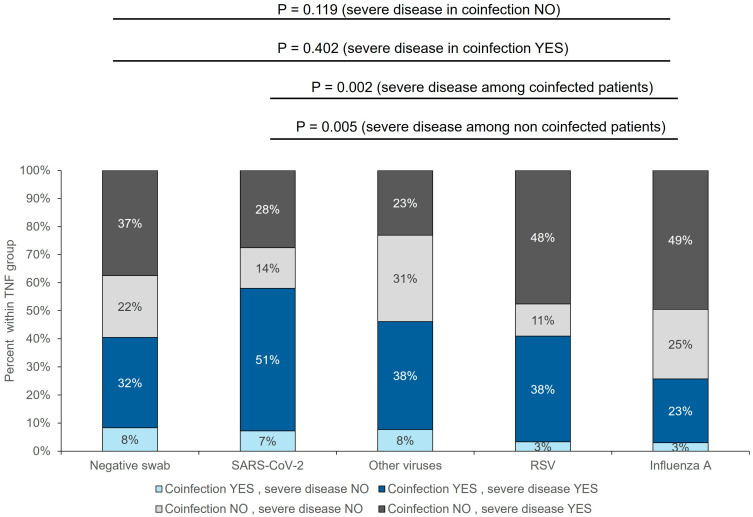
Prevalence of severe disease with and without a coinfection in patients with different viral isolates. Percentages are within group.

Anthropometric, comorbidities, and home treatments in coinfected patients were homogeneous across different viral infections (Supplementary Table S3). In patients with *Influenza*, coinfection was associated more frequently with lung infiltrates, lower blood pressure, and a significantly higher chance of being exposed to non-invasive or invasive respiratory support (Supplementary Table S4). Patients with *Influenza* and SARS-CoV-2 presented more frequently with a severe disease if coinfected (87.5% vs. 65.5%, *p* = 0.042, and 88.5% vs. 66.7%, *p* = 0.040, respectively), while the presence of severe disease was independent of coinfection in patients with RSV and other viruses (Supplementary Table S4). The highest prevalence of severe disease in patients with coinfection was observed in COVID-19 patients (51%), while the lowest was in patients with *Influenza* (23%) (Figure 5). ICU admission and in-hospital and ICU mortality were independent of the presence of coinfection in patients with COVID-19, RSV, and other viruses, while significantly higher in coinfected patients with Influenza (19.2% vs. 1.3%, *p* = 0.004).

### 3.6. Prevalence and Burden of MDR Coinfections

The prevalence of MDR coinfections was 12.5% (81/652), equal to 30.8% of all coinfections. The most frequent MDR bacteria were ESBL-positive Enterobacteriaceae (49%) and carbapenem-resistant Gram-negative bacteria (20%) (Supplementary Figure S3). The prevalence of MDR infections was not different in patients with and without a viral isolate (33% (34/103) and 29% (47/160)). The prevalence of MDR coinfections significantly differed between viral groups, being highest in patients with SARS-CoV-2 (40%; Supplementary Figure S4), while the distribution of MDR species did not significantly differ between groups (Supplementary Figure S5). An MDR coinfection was associated with CPAP support, a higher ICU-related and in-hospital mortality (Supplementary Table S5).

## 4. Discussion

The present study demonstrated that the prevalence of coinfections in unselected patients presenting to the emergency department with flu-like symptoms and/or respiratory failure and a positive viral swab is as high as 40%, with SARS-CoV-2 and RSV having the highest proportion of coinfected patients. Overall, coinfected patients were at higher risk of presenting with severe disease, respiratory failure, and need for non-invasive or invasive respiratory support, resulting in a significantly longer length of hospital stay, higher exposure to ICU, in-hospital complications, and a higher in-hospital mortality.

Secondary bacterial pneumonia complicating a viral infection is known to be associated with unfavorable outcomes in adult patients with Influenza virus and SARS-CoV-2 infection [14,15,16]. Large series published during the COVID-19 pandemic have demonstrated that bacterial coinfections in patients with SARS-CoV-2 pneumonia, although inhomogeneous, were very common and characterized by worse clinical outcomes and the highest mortality [15,16].

Our data are in line with the prospective study by Luchsinger and colleagues, which systematically tested patients with pneumonia for viral and/or bacterial isolates, finding a coinfection prevalence of 17% [23], very similar to the 16% found in our study, when also including patients without lung infiltrates. However, the same authors did not observe an association between clinical severity and coinfection, supposedly because of the limited number of patients included [23].

A large cohort from Hong Kong including more than 19,000 patients with a viral and/or bacterial respiratory infection recently showed coinfection rates of 6.8% [12]. However, the study was conducted before the COVID-19 pandemic and analyzed only positive cultures within 48 h of admission, partially justifying the difference in our findings [12]. However, consistent with our results, the authors demonstrated that patients with coinfection had a higher rate of ICU admission and mortality compared with patients with a bacterial infection alone [12].

We showed that in-hospital mortality in coinfected patients reached 16.5% compared with patients with an isolated viral infection (3.2%) and patients without coinfection (8.0%). To date, data on characteristics, risk factors, and outcomes associated with bacterial coinfections also in patients without CAP have been limited. The present study demonstrated that lower and upper respiratory viral infections can both pose patients at risk of coinfection and unfavorable outcomes at least as much as observed in patients with viral CAP. Indeed, previous retrospective studies conducted in patients with viral pneumonia showed how bacterial coinfection was associated with reduced survival in patients admitted with RSV infection, with consistently higher rates of coinfection compared with *Influenza* [23,24,25]. We confirmed that patients with RSV infection were more prone to have a coinfection and to present with severe disease compared with *Influenza*. Moreover, the time to develop a secondary infection was lower in patients with RSV compared with patients with *Influenza*.

In terms of risk factors for viral/bacterial coinfection, the majority of the literature focused on patients hospitalized with COVID-19 pneumonia [26,27,28,29], with heterogeneous results. Lopez-Herrero and coworkers indicated organ failure, male sex, and obesity as major risk factors for bacterial coinfection in COVID-19 patients [27]. Severe and critical COVID-19 together with cardiovascular disease were the only variables associated with bacterial coinfections in a retrospective Chinese cohort [27]. Our model showed that nephropathy, COVID-19 infection per se, the absence of COVID-19 vaccination, and lower blood pressure represent independent risk factors for coinfection in patients with a positive viral swab. Chronic kidney disease is an established risk factor for CAP and hospital-acquired infections, and it is also a risk factor for more severe disease, cardiovascular complications, and mortality [30]. Moreover, several comorbidities and immune suppression can lead to severe complications in patients with kidney disease who acquire a viral or bacterial infection, especially in the case of COVID-19 [31]. In our study, patients with SARS-CoV-2 had the highest rate of coinfections and severe disease among coinfected patients; SARS-CoV-2 infection was also associated with the highest prevalence of MDR pathogens (40% of coinfected patients). SARS-CoV-2 infection modulates the immune response mediated by IFNα/β, TLR-signaling, and different cytokine-related macrophage recruitment, leading to the suppression of anti-bacterial host defenses, thus favoring bacterial infections and also promoting severe forms of disease [32]. Different mechanisms may predispose to coinfection in patients with a primary viral infection. In *influenza* infection models, increased accumulation of dysfunctional neutrophils with impaired bacterial clearance and tissue damage, virus-induced type 1 Interferon signaling, interleukin-13-mediated type 2 innate lymphoid cell protective effects, and infection-related modulation of type 17 immunity, were all suggested to play a role in the modulation of innate and adaptive immune responses in secondary bacterial infections [10]. In COPD patients with *Rhinovirus* infection and no evidence of bacterial infection, Mallia and colleagues observed the occurrence of a secondary bacterial infection in 60% of cases. The latter was associated with higher viral load and more severe respiratory symptoms, demonstrating the link between viral and bacterial infections in COPD exacerbations [33]. The absence of bacteria before the viral infection drives the hypothesis that a secondary bacterial infection might arise both from an exogenous infection or from an overgrowth of resident bacteria secondary to virus-mediated immune depression [33].

The present study showed a relatively high incidence of Enterobacteriaceae and *E. coli* coinfections, which was probably secondary to the study design and patients’ baseline characteristics. In fact, both bacterial/fungal co- and super-infections were considered in patients consecutively enrolled from the emergency department with a viral respiratory tract infection. We hypothesize that the epidemiology of bacterial coinfections in our study could be driven by the severity of infection (on average > 65% of our patients had a severe disease including respiratory failure, sepsis, need for invasive or non-invasive mechanical ventilation, and ICU admission) and by a high prevalence of elderly patients with co-morbidities in our sample. In the literature, data on co-infection epidemiology are heterogeneous and largely depend upon care setting, sampling methodology, and protocols. A clinical review including >2000 patients with Influenza-, MERS-, or COVID-19-related pneumonia reported that Enterobacteriaceae were responsible for 18% of secondary bacterial infections, while *S. pneumoniae* was found in 13% of cases [15]. In a retrospective study by Chen and colleagues [34] in ICU patients with viral pneumonia, co-infection with Enterobacteriaceae was more prevalent in patients with COVID-19 pneumonia, while E. coli was mainly responsible for ICU-acquired super-infections. Accordingly, the most frequent cause of coinfection in patients with Influenza pneumonia was *S. aureus* and *P. aeruginosa*, while Enterococci were found mainly in patients with ICU super-infections (16.7%) [34]. Finally, the prevalence of Enterobacteriaceae and *E. coli* co-infection in patients with COVID-19 pneumonia was 3.9% and 4.4%, respectively, in a recent meta-analysis [35], while Legionella pneumophila, *H. influenzae*, *M catarrhalis*, and *S. pneumoniae* accounted for 4.4%, 6.5%, 4.3%, and 7.8% of co-infections, respectively. Data from national/regional microbiological surveillance for outpatient bacterial infections other than TB are currently lacking in Italy. Indeed, our results are reflected by the surveillance data from the Italian National Center for Prevention and Disease Control on hospital-acquired infections, which demonstrated that the most frequently isolated pathogens in 2022 were *E. coli* (11.7%), *K. pneumoniae* (11.6%), and *P. aeruginosa* (8.2%) [36]. Moreover, hospital-acquired infections peaked in the seventh to eighth decade, justifying the high incidence of co- and super-infections in our study [36]. Data from the literature indicate that in Italy, the incidence of carbapenem-resistant organisms, VRE, MRSA, and *E. coli* have significantly increased over the last 5 years, a trend that is reflected by our results, especially in terms of MDR-resistant bacteria [37].

Interestingly, the absence of COVID-19 vaccination impacted negatively on the risk of acquiring a coinfection. This could be explained by two hypotheses. First, non-vaccinated patients were more prone to acquiring COVID-19 infection, thus dragging the infection-related risk of developing a coinfection. Second, we speculate that the presence of COVID-19 vaccination in some patients might have promoted an adaptive cross-reactive immune response that might have favored the protection against secondary infections. It should be noted that viral vaccines, by means of herd immunity, reducing the risk of secondary bacterial infection and also reducing the chance of developing virus-mediated upper and lower respiratory tract infections, might also limit the use of inappropriate antibiotic treatments, positively affecting antimicrobial resistance [38].

We think that clinical step-up modeling of risk factors might be of help for clinical decision-making, especially in the emergency setting, when often inappropriate preventive antimicrobial treatments are applied.

To our knowledge, this is one of the few studies that investigated the prevalence of MDR coinfections outside the ICU in patients with community-acquired viral infections. We observed that MDR coinfections varied from 24% in patients with RSV to 40% in patients with COVID-19. MDR coinfections are associated with very high mortality rates, especially in the case of MRSA and ESBL isolates, as demonstrated by large retrospective cohorts [12]. This is in line with our observations. In fact, we report significantly higher ICU and in-hospital mortality (26.5% vs. 8.5%, *p* = 0.031) in patients with a viral-positive swab compared with patients without a viral infection. MDR isolates were associated with a worse clinical presentation but were similar across viral infection groups, indicating that the presence of MDR pathogens should be suspected in patients with severe disease with risk factors for coinfection. Unfortunately, we were unable to assess specific risk factors for MDR coinfections due to the small number of cases.

### Study Limitations

The present study has several limitations that need to be discussed. First, this is a single-center study that, although conducted in a hospital with a large catching area, might suffer from selection bias and, therefore, a lack of result generalizability. In fact, despite enrolling consecutive patients in a real-life setting, the average age and the prevalence of elderly patients with a high prevalence of comorbidities (mainly COPD) might have influenced the prevalence of severe disease and study outcomes, thus contributing to the results’ reproducibility in different regions and settings. Patients’ characteristics and the study design, which included both co- and super-infections, might also be responsible for a higher-than-expected rate of coinfections driven by Enterobacteriaceae and *E. coli*, with a lower-than-expected prevalence of *S. pneumoniae*, *H. influenzae*, and *L. pneumophila*, which probably reflect local nosocomial bacterial epidemiology and should be considered with caution when comparing studies conducted in other countries and clinical settings. Moreover, the prevalence of viral pathogens might not be accurate, considering RSV and *Influenza* seasonal fluctuations. Indeed, the study period of interest (2022–2023) exactly covered the peak of ILI for that season, as demonstrated by the Epidemiological Report of RespiVirNet, the surveillance organ for respiratory viruses of the Italian Health Institute [39]. Third, we did not collect data on *Influenza* or pneumococcal vaccination, which were largely lacking in the patients’ records, and this might have caused reporting bias. This study has also strengths that should be highlighted, which consist of a very accurate and detailed patient characterization that includes both the clinical history and data on the clinical presentation, in-hospital complications, and outcomes. Moreover, the per-protocol viral testing allowed us to include a large number of patients, avoiding an important confounding element such as testing bias, often present in previous studies published on the topic.

## 5. Conclusions

Patients with respiratory symptoms and a viral infection are at high risk of bacterial coinfections, which carry a significant morbidity and mortality burden compared with a bacterial or viral infection alone. The evaluation of specific risk factors should drive clinical decision-making in terms of the need for monitoring, testing for bacterial infections, and possible targeted antibiotic treatments. In this view, the high prevalence of coinfections with MDR pathogens stresses the need for careful antibiotic stewardship to avoid the introduction of unnecessary and ineffective treatments. Indeed, the results of this study highlight the pivotal role of vaccination and should foster epidemiological considerations in terms of its collateral and adjunctive benefits, especially in patients at risk. Larger multicenter international studies including a wider age and frailty patients’ spectra should be promoted to better define the clinical, geographical, seasonal, and ethnical determinants of risk factors for coinfections in patients with viral infections and patterns of antibiotic resistance in patients at risk of MDR pathogens.

## Figures and Tables

**Table 1:** Anthropometric variables, comorbidities, and home treatments in the study cohort.

	Viral Swab-Tested Patients (N = 652)				
Variable	Viral Swab Positive with Coinfection (A) N = 103	Viral Swab Positive Without Coinfection (B) N = 154	Viral Swab Positive Without Coinfection and Viral Swab Negative (C) N = 549	*p*-Value (A) vs. (B)	*p*-Value (A) vs. (C)
Males, n (%)	58 (56.3)	77 (50.0)	282 (51.4)	0.373	0.391
Age, years	80 (65–88)	74 (58–83)	74 (59–83)	**<0.001**	**<0.001**
Age ≥70 years, n (%)	71 (68.9)	93 (60.4)	325 (59.2)	0.103	**0.039**
Hospitalizations last 12 months	0 (0–1)	0 (0–0)	0 (0–1)	0.050	0.902
Hospitalizations ≥ 2, n (%)	8 (7.8)	7 (4.5)	54 (9.8)	0.395	0.211
Nursing home resident, n (%)	4 (3.9)	3 (1.9)	25 (4.6)	0.443	1.000
COVID-19 vaccination, n (%)	64 (78.0)	113 (95.8)	382 (69.6)	**<0.001**	**<0.001**
**Comorbidities**					
Charlson, score	4 (2–6)	4 (2–6)	4 (2–6)	0.554	0.536
Previous COVID-19, n (%)	20 (19.4)	22 (14.3)	89 (16.2)	0.304	0.471
Obesity, n (%)	4 (3.9)	12 (11.6)	63 (11.5)	0.159	**0.011**
Chronic heart failure, n (%)	24 (23.8)	19 (12.3)	102 (18.7)	**0.025**	0.274
Ishemic heart disease, n (%)	21 (20.6)	18 (11.7)	97 (17.7)	0.075	0.486
Stroke, n (%)	12 (11.8)	11 (7.2)	44 (8.1)	0.265	0.249
Arterial hypertension, n (%)	56 (54.9)	86 (55.8)	317 (58)	0.898	0.587
Atrial fibrillation, n (%)	30 (29.4)	32 (20.8)	111 (20.2)	0.136	**0.049**
Arrhytmia (other), n (%)	4 (3.9)	9 (5.8)	39 (7.1)	0.573	0.284
Valvulopathy, n (%)	22 (21.6)	25 (16.2)	100 (18.2)	0.323	0.410
Vasculopathy, n (%)	15 (14.7)	24 (15.6)	101 (18.4)	1.000	0.402
CVD (any), n (%)	73 (71.6)	107 (69.5)	392 (71.5)	0.781	1.000
Diabetes, n (%)	22 (21.6)	36 (23.4)	149 (27.1)	0.763	0.271
Ulcer, n (%)	3 (2.9)	2 (1.3)	19 (3.5)	0.390	1.000
Nephropathy, n (%)	20 (19.6)	13 (8.4)	78 (14.2)	**0.013**	0.175
Immunocompromized, n (%)	14 (13.7)	11 (7.1)	40 (7.3)	0.090	**0.048**
Epatopathy, n (%)	10 (9,8)	8 (5.2)	40 (7.3)	0.212	0.416
Dementia, n (%)	16 (15.7)	17 (11.0)	74 (13.5)	0.341	0.534
Emiplegy, n (%)	6 (5.9)	4 (2.6)	10 (1.8)	0.203	**0.027**
Psychiatric disorders, n (%)	7 (6.9)	17 (11.0)	60 (10.9)	0.284	0.286
Rheumatologic disorders, n (%)	9 (8.8)	8 (5.2)	28 (5.1)	0.308	0.159
Solid tumors, n (%)	11 (10.8)	22 (14.3)	83 (15.1)	0.452	0.286
Leukemia, n (%)	3 (2.9)	7 (4.5)	11 (2.0)	0.744	0.469
Lymphoma, n (%)	4 (3.9)	6 (3.9)	12 (2.2)	1.000	0.295
AIDS, n (%)	5 (4.9)	0 (0)	10 (1.8)	**0.009**	0.070
Ex/active smoke, n (%)	34 (33.0)	50 (32.5)	161 (29.3)	0.402	0.399
COPD, n (%)	24 (23.5)	30 (19.6)	127 (23.3)	0.532	1.000
Asthma, n (%)	5 (4.9)	14 (9.2)	45 (8.2)	0.233	0.314
Bronchiectasis, n (%)	4 (3.9)	3 (2.0)	15 (2.7)	0.442	0.521
Interstitial lung disease, n (%)	3 (2.9)	4 (2.6)	12 (2.2)	1.000	0.717
**Home treatment**					
LTOT, n (%)	8 (7.8)	11 (7.2)	49 (9.0)	1.000	0.850
Bronchodilators, n (%)	19 (18.6)	31 (20.3)	113 (20.7)	0.872	0.689
ICS, n (%)	17 (16.7)	24 (15.7)	89 (16.3)	0.863	0.885
Chronic steroid therapy, n (%)	2 (2.0)	6 (3.9)	30 (5.5)	0.482	0.209
Immunosuppressants, n (%)	4 (4.0)	7 (4.6)	18 (3.3)	1.000	0.764

Patients with a confirmed viral infection are grouped into patients with (column A) or without (column B) a coinfection. Patients with a positive viral swab and a coinfection were compared with the rest of the tested patients (column C). Data are reported as median (interquartile range) if not stated otherwise. AIDS = Acquired Immune Deficiency Syndrome; COPD = Chronic Obstructive Pulmonary Disease; COVID-19 = Coronavirus 2019 Disease; CVD = cardiovascular disease; ICS = inhaled corticosteroids; LTOT = long-term oxygen therapy. Statistically significant differences are highlighted in bold.

**Table 2:** Characteristics at emergency department presentation, in-hospital treatments, and clinical outcomes in the study cohort.

	Viral Swab-Tested Patients (N = 652)				
Variable	Viral Swab Positive with Coinfection (A) N = 103	Viral Swab POSITIVE without Coinfection (B) N= 154	Viral Swab Positive Without Coinfection and Viral Swab Negative (C) N = 549	*p*-Value (A) vs. (B)	*p*-Value (A) vs. (C)
**Isolated pathogens**					
SARS-CoV-2, n (%)	40 (38.8)	29 (18.8)	29 (5.3)	**<0.001**	**<0.001**
Influenza A, n (%)	26 (25.2)	75 (48.7)	75 (13.7)	**<0.001**	**0.003**
RSV, n (%)	25 (24.3)	36 (23.4)	36 (6.6)	0.492	**<0.001**
Other viruses, n (%)	12 (11.6)	14 (9.1)	14 (2.5)	0.321	**<0.001**
**Imaging**					
Chest X-ray infiltrates, n (%)	44 (43.6)	40 (27.2)	157 (29.8)	**0.009**	**0.010**
Lobar pneumonia, n (%)	17 (16.8)	18 (12.1)	90 (17)	0.353	**1.000**
Bilateral pneumonia, n (%)	26 (25.7)	20 (13.4)	53 (8.4)	**0.019**	**<0.001**
Interstitial pneumonia, n (%)	29 (28.7)	27 (18.1)	56 (10.6)	0.063	**<0.001**
Pleural effusion, n (%)	14 (13.9)	13 (8.7)	86 (16.3)	0.217	0.656
**Clinical presentation**					
Respiratory rate, bpm	21 (19–28)	22 (18–26)	20 (18–26)	0.758	0.279
Respiratory rate ≥ 24 bpm, n (%)	30 (42.3)	38 (42.2)	135 (24.6)	0.562	0.352
SBP, mmHg	130 (120–142)	140 (125–150)	131 (120–150)	**0.007**	0.281
SBP ≤ 130 mmHg, n (%)	58 (56.3)	57 (37.0)	258 (47.0)	**0.002**	0.068
SBP ≤ 100 mmHg, n (%)	6 (6.1)	5 (3.4)	29 (5.3)	0.251	0.502
Heart rate, beats/min	88 (78–108)	90 (80–101)	90 (78–101)	0.569	0.754
Heart rate ≥100 beats/min, n (%)	30 (30.6)	47 (31.1)	168 (30.6)	0.523	0.443
Fever, n (%)	55 (55.0)	88 (57.5)	242 (45.2)	0.699	0.081
Confusion, n (%)	23 (23.7)	24 (15.9)	97 (18.1)	0.137	0.206
Acute respiratory failure, n (%)	60 (58.3)	76 (49.4)	257 (46.8)	0.202	**0.041**
PaO2/FiO2, mmHg	268 (224–313)	276 (233–326)	276 (227–322)	0.417	0.579
Type 1 aRF, n (%)	46 (44.7)	59 (38.3)	196 (35.7)	0.365	0.096
Type 2 aRF, n (%)	12 (11.7)	18 (11.7)	65 (11.8)	1.000	1.000
Sodium, mmol/L	137 (134–140)	138 (135–139)	138 (135–140)	0.952	0.283
Glicaemia, mg/dL	122 (99–167)	116 (103–140)	118 (100–151)	0.565	0.577
CRP, mg/L	75 (36–162)	53 (23–117)	62 (22–138)	**0.026**	0.064
CRP ≤ 20 mg/L, n (%)	14 (13.6)	33 (21.4)	127 (23.1)	0.078	**0.024**
White blood cells, cells/µL	9020 (7128–13.553)	8050 (5758–10,833)	9820 (7080–12,970)	**0.010**	0.760
WBC ≥ 8000 cells/µL, n (%)	65 (63.7)	78 (52)	364 (66.3)	**0.043**	0.311
Urea, mmol/L	53 (37–95)	46 (36–62)	45 (32–69)	0.160	0.095
Creatinine, µmol/L	0.96 (0.76–1.63)	0.91 (0.72–1.15)	0.94 (0.73–1.30)	0.074	0.320
Aspartate transaminase, IU/L	19 (11–35)	21 (15–34)	20 (13–33)	0.143	0.438
Shock, n (%)	9 (8.8)	1 (0.7)	20 (3.6)	**0.001**	**0.033**
Sepsis, n (%)	13 (12.7)	1 (0.7)	47 (8.6)	**<0.001**	0.195
**In-hospital treatment**					
Antibiotics, n (%)	88 (87.1)	102 (67.5)	419 (77.9)	**<0.001**	**0.044**
Systemic corticosteroids, n (%)	66 (65.3)	82 (54.7)	248 (46.6)	0.116	**<0.001**
Inhaled corticosteroids, n (%)	46 (45.5)	82 (54.7)	241 (45.3)	0.160	**1.000**
Oseltamivir, n (%)	20 (19.4)	52 (33.8)	60 (10.9)	**0.016**	**0.021**
Other antivirals, n (%)	30 (29.1)	16 (10.5)	34 (6.3)	**<0.001**	**<0.001**
**Respiratory support**					
Oxygen therapy, n (%)	85 (82.5)	103 (66.9)	360 (65.6)	**0.006**	**<0.001**
HFNC, n (%)	17 (16.5)	5 (3.2)	38 (6.9)	**<0.001**	**0.003**
CPAP, n (%)	17 (16.5)	11 (7.1)	35 (6.4)	**0.024**	**0.001**
NIV, n (%)	14 (13.6)	10 (6.5)	41 (7.5)	0.079	0.052
ETI, n (%)	17 (16.5)	5 (3.2)	27 (4.9)	**<0.001**	**<0.001**
**Outcomes**					
Length of stay, days	25 (14–37)	8 (3–13)	10 (5–18)	**<0.001**	**<0.001**
Time to coinfection, days	3 (2–9)	-	5 (2–9)	-	0.391
Severe disease, n (%)	91 (88.3)	104 (67.5)	379 (69.0)	**<0.001**	**<0.001**
Time to ICU admission, days	3 (1–5)	1 (1–5)	1 (0–4)	0.250	0.268
ICU, n (%)	21 (20.4)	6 (3.9)	29 (5.3)	**<0.001**	**<0.001**
Tracheostomy, n (%)	11 (10.8)	1 (0.6)	9 (1.6)	**<0.001**	**<0.001**
In-hospital death, n (%)	17 (16.5)	5 (3.2)	44 (8.0)	**<0.001**	**0.010**
ICU death, n (%)	17 (16.5)	5 (3.2)	44 (8.0)	**<0.001**	**0.010**

Patients with a confirmed viral infection are grouped into patients with (column A) or without (column B) a coinfection. Patients with a positive viral swab and a coinfection were compared with the rest of the tested patients (column C). Data are reported as median (interquartile range) if not stated otherwise. aRF = acute respiratory failure; CRP = C reactive protein; ICU = Intensive Care Unit; ETI = Endotracheal Intubation; NIV = non-invasive ventilation; CPAP = continuous positive airway pressure; HFNC = high flow nasal cannula; ICS = inhaled corticosteroids; FiO2 = fractional inhaled oxygen; PaO2 = arterial partial pressure of oxygen; RSV = Respiratory Syncytial Virus; SARS-CoV-2 = severe acute respiratory syndrome coronavirus 2; SBP = systolic blood pressure; WBC = white blood cell count. Statistically significant differences are highlighted in bold.

**Table 3:** Risk factors for coinfection in patients with acute viral infection.

Model A Baseline Characteristics				Model B Clinical Presentation				Model C Biomarkers (Continuous)				Model D Biomarkers (Categorical ^†^)			
Variable	OR	95% CI	*p*-Value	Variable	OR	95% CI	*p*-Value	Variable	OR	95% CI	*p*-Value		OR	95% CI	*p*-Value
Heart failure	1.929	0.789–4.719	0.150		1.956	0.777–4.926	0.155		2.293	0.845–6.225	0.103		2.357	0.918–6.048	0.075
Nephropathy	**3.649**	**1.422–9.367**	**0.007**		**3.086**	**1.161–8.208**	**0.024**		2.792	0.959–8.127	0.060		**3.193**	**1.098–9.291**	**0.033**
COVID vaccination	**0.160**	**0.055–0.471**	**<0.001**		**0.163**	**0.054–0.489**	**0.001**		**0.175**	**0.055–0.557**	**0.003**		**0.155**	**0.047–0.504**	**0.002**
Age (cont.)	1.016	0.993–1.040	0.175		1.011	0.987–1.035	0.386		1.012	0.986–1.038	0.377	Age ≥ 70 years	0.943	0.439–2.025	0.880
				Bilateral pneumonia	1.321	0.572–3.047	0.907		1.140	0.444–2.923	0.785		1.239	0.497–3.089	0.646
				COVID infection	**2.390**	**1.165–4.902**	**0.017**		**2.356**	**1.080–5.140**	**0.031**		**2.795**	**1.292–6.046**	**0.009**
								CRP	1.000	0.996–1.004	0.983	CRP	1.002	0.997–1.006	0.460
								SBP	**0.980**	**0.967–0.995**	**0.007**	SBP ≤ 130 mmHg	**3.588**	**1.776–7.251**	**<0.001**
								WBC	1.000	1.000–1.000	0.075	WBC ≥ 8000 cells/µL	1.668	0.799–3.480	0.173

Logistic regression analysis for the prediction of risk factors for the occurrence of coinfection in patients with a positive viral swab. Models depending on the available clinical information and biomarkers are shown. Significant variables are in bold. WBCs: white blood cells; CRP = C-reactive protein; SBP = systolic blood pressure. † C-reactive protein was left as a continuous variable as no physiologically or pathologically sound threshold was identified to be considered as a risk factor to differentiate the presence of coinfection. Significant risk factors are highlighted in bold.

## Data Availability

The data that support the findings of this study are available from the corresponding author, PS, upon reasonable request.

## Supplementary Materials

The following supporting information can be downloaded at: https://www.mdpi.com/article/10.3390/pathogens13110993/s1, Figure S1: Distribution of bacterial coinfections in the study cohort. Numbers refer to the prevalence of the single pathogen; Figure S2: Distribution of bacterial coinfections in patients with (B) and without (A) a viral isolate. Numbers refer to the prevalence of the single pathogen; Figure S3: Distribution of multi-drug resistant (MDR) coinfections in the whole study cohort. * = 9 patients had a positive blood culture and 3 patients had a positive urine culture for VRE (Vancomycin-Resistant Enterococci); Figure S4: Prevalence of MDR coinfections in patients with a coinfection. Percentages are within group; Figure S5: Distribution of MDR pathogens for each viral isolate group. Numbers refer to patients with a specific MDR pathogen.
