# Supplementary material for: Burden and Risk Factors for Coinfections in Patients with a Viral Respiratory Tract Infection
Source: Pathogens. 2024 Nov 13;13(11):993. doi: 10.3390/pathogens13110993 (PMC11597400; doi:10.3390/pathogens13110993)
Supplement: Supplementary file 1 [file pathogens-13-00993-s001.zip › Supplementary Table S1_Microorganisms and coinfections.pdf]

Table S1. Distribution of bacterial/fungal isolates in patients with and without a viral infection.

Microorganism	All patients N = 654	Swab – N = 395	Swab + N = 257	p-value
No coinfections, n (%)	389 (59.7)	235 (59.5)	154 (59.9)	0.935
Streptococcus pneumoniae, n (%)	15 (2.3)	7 (1.8)	8 (3.1)	0.292
Escherichia coli, n (%)	61 (9.4)	38 (9.6)	23 (8.9)	0.891
Legionella pneumophila, n (%)	3 (0.5)	2 (0.5)	1 (0.4)	1.000
Enterobacteriaceae, n (%)	35 (5.4)	22 (5.6)	13 (5.1)	0.860
Klebsiella spp, n (%)	20 (3.1)	13 (3.3)	7 (2.7)	0.818
Pseudomonas aeruginosa, n (%)	9 (1.4)	4 (1.0)	5 (1.9)	0.327
MSSA, n (%)	19 (2.9)	11 (2.8)	8 (3.1)	0.815
MRSA, n (%)	5 (0.8)	3 (0.8)	2 (0.8)	1.000
Staphilococci (other), n (%)	34 (5.2)	20 (5.1)	14 (5.4)	0.858
Gram negative (other), n (%)	15 (2.3)	6 (1.5)	9 (3.5)	0.113
Anaerobes, n (%)	2 (0.3)	1 (0.3)	1 (0.4)	1.000
Mycobacterium tuberculosis, n (%)	1 (0.2)	0 (0)	1 (0.4)	0.394
Aspergillus spp, n (%)	1 (0.2)	1 (0.3)	0 (0)	1.000
Candida spp, n (%)	27 (4.1)	21 (5.3)	6 (2.3)	0.071
Other bacteria, n (%)	16 (2.5)	11 (2.8)	5 (1.9)	0.610

spp = species. MRSA = meticillin resistant Staphylococcus aureus; MSSA = meticillin-sensitive Staphylococcus aureus.
