# Supplementary material for: Burden and Risk Factors for Coinfections in Patients with a Viral Respiratory Tract Infection
Source: Pathogens. 2024 Nov 13;13(11):993. doi: 10.3390/pathogens13110993 (PMC11597400; doi:10.3390/pathogens13110993)
Supplement: Supplementary file 1 [file pathogens-13-00993-s001.zip › Supplementary Table S2_Microorganisms and coinfections depending on virus.pdf]

Table S2. Distribution of bacterial/fungal isolates in patients with coinfection and different viral infections.

**Microorganism**	**Viral swab + N = 257**	**Influenza A (A) N = 101**	**RSV (B) N = 61**	**SARS-CoV-2 N = 69**	**Other viruses N = 26**	**p-value**	**p-value (A vs B)**
Streptococcus pneumoniae, n (%)	8 (3.1)	4 (4)	1 (1.6)	2 (2.9)	1 (3.8)	0.693	0.376
Escherichia coli, n (%)	23 (8.9)	6 (5.9)	4 (5.9)	11 (15.9)	2 (7.7)	0.231	0.561
Legionella pneumophila, n (%)	1 (0.4)	0 (0)	1 (1.6)	0 (0)	0 (0)	0.596	0.377
Enterobacteriaceae, n (%)	13 (5.1)	3 (3.0)	3 (0.5)	7 (10.1)	0 (0)	0.217	0.406
Klebsiella spp, n (%)	7 (2.7)	0 (0)	2 (3.3)	4 (5.8)	1 (3.8)	0.282	0.140
Pseudomonas aeruginosa, n (%)	5 (1.9)	0 (0)	2 (3.3)	3 (4.3)	0 (0)	0.083	0.140
MSSA, n (%)	8 (3.1)	1 (1.0)	2 (3.3)	4 (5.8)	1 (3.8)	0.481	0.317
MRSA, n (%)	2 (0.8)	1 (1.0)	1 (1.6)	0 (0)	0 (0)	0.842	0.613
Staphilococci (other), n (%)	14 (5.4)	6 (5.9)	3 (4.9)	3 (4.3)	2 (7.7)	0.967	0.542
Gram negative (other), n (%)	9 (3.5)	2 (2.0)	2 (3.3)	1 (1.4)	4 (15.4)	**<0.001**	0.485
Anaerobes, n (%)	1 (0.4)	1 (1.0)	0 (0)	0 (0)	0 (0)	0.725	0.623
Mycobacterium tuberculosis, n (%)	1 (0.4)	1 (1.0)	0 (0)	0 (0)	0 (0)	0.243	0.623
Aspergillus spp, n (%)	0 (0)	0 (0)	0 (0)	0 (0)	0 (0)	0.957	-
Candida spp, n (%)	6 (2.3)	0 (0)	3 (4.9)	2 (2.9)	1 (3.8)	0.191	0.052
Other bacteria, n (%)	5 (1.9)	1 (1.0)	1 (1.6)	3 (4.3)	0 (0)	0.568	0.613

Statistically significant values are highlighted in bold.
