# Supplementary material for: Burden and Risk Factors for Coinfections in Patients with a Viral Respiratory Tract Infection
Source: Pathogens. 2024 Nov 13;13(11):993. doi: 10.3390/pathogens13110993 (PMC11597400; doi:10.3390/pathogens13110993)
Supplement: Supplementary file 1 [file pathogens-13-00993-s001.zip › Supplementary Table S3_Patients characteristics by rivus group.pdf]

	SARS-CoV-2 infection (N = 69)			Influenza A infection (N = 101)			RSV infection (N = 61)			Other viruses infection (N = 26)		
	With coinfection (N = 40)	*Without coinfection (N = 29)*	*p-value*	*With coinfection (N = 26)*	*Without coinfection (N = 75)*	*p-value*	*With coinfection (N = 25)*	*Without coinfection (N = 36)*	*p-value*	*With coinfection (N = 12)*	*Without coinfection (N = 14)*	*p-value*
**Characteristics**												
Males, n (%)	21 (52.5)	19 (65.5)	0.329	17 (65.4)	38 (50.7)	0.254	11 (44)	15 (41.7)	1.000	9 (75.0)	5 (35.7)	0.062
Age, years	84 (73-92)	78 (69-84)	**0.047**	76 (65.5-87.5)	69 (57-80)	**0.022**	81 (62.5-88)	80 (70-85)	0.730	69.5 (54.7-86.5)	72 (26.5-86)	0.595
Age ≥ 70 years, n (%)	32 (80.0)	22 (75.9)	0.771	17 (65.4)	37 (49.3)	0.178	16 (64.0)	27 (75.0)	0.401	6 (50.0)	7 (50.0)	1.000
Nursing home residents, n (%)	4 (10)	0 (0)	0.133	0 (0)	1 (7.1)	1.000	0 (0)	1 (2.8)	1.000	0 (0)	1 (7.1)	1.000
Hospitalizations, n (%)	0 (0-1)	0 (0-0)	0.691	0 (0-1)	0 (0-0)	0.200	0 (0-1)	0 (0-1)	0.950	0 (0-1)	0 (0-0)	0.231
Hospitalization ≥ 1/year, n (%)	12 (30.0)	6 (20.7)	0.412	6 (25.0)	12 (16.4)	0.372	9 (15.3)	11 (32.4)	0.788	5 (41.7)	2 (14.3)	0.190
Hospitalization ≥ 2/year, n (%)	3 (7.5)	1 (3.4)	0.634	3 (12.5)	4 (5.5)	0.359	1 (1.7)	3 (5.1)	0.630	0 (0)	0 (0)	-
COVID vaccination, n (%)	25 (71)	24 (100)	**0.004**	4 (80)	8 (100)	0.385	19 (90.5)	23 (88.5)	1.000	4 (80)	8 (100)	0.385
Smoker (ex/current), n (%)	10 (33.3)	5 (26.3)	0.754	8 (44.4)	27 (45.8)	1.000	13 (61.9)	16 (48.5)	0.407	3 (37.5)	2 (20.0)	0.608
**Comorbidities**												
Charlson index, score	3 (2-6)	4 (2.5-6)	0.664	4 (2-6)	4 (2-6)	0.475	4 (1-5)	4 (1-6)	0.630	4.5 (2.2-7.5)	3.5 (2.0-5.2)	0.347
Previous COVID-19, n (%)	5 (12.5)	5 (17.2)	0.732	3 (25)	1 (7.1)	0.306	6 (24)	5 (13.9)	0.333	3 (25)	1 (7.1)	0.306
Heart failure, n (%)	12 (30)	3 (10.3)	0.076	3 (25)	4 (28.6)	1.000	5 (20.8)	10 (27.8)	0.762	3 (25)	4 (28.6)	1.000
Ischemic heart disease, n (%)	9 (22.5)	3 (10.3)	0.218	2 (16.7)	0 (0)	0.203	4 (16)	6 (16.7)	1.000	2 (16.7)	0 (0)	0.203
Cerebrovascular, n (%)	4 (10)	2 (6.9)	1.000	1 (8.3)	1 (7.1)	1.000	4 (16)	4 (11.4)	0.708	1 (8.3)	1 (7.1)	1.000
Arterial hypertension, n (%)	18 (45)	18 (62.1)	0.223	7 (58.3)	8 (57.1)	1.000	16 (64)	20 (55.6)	0.601	7 (58.3)	8 (57.1)	1.000
Atrial fibrillation, n (%)	14 (35)	7 (24.1)	0.430	1 (8.3)	3 (21.4)	0.598	9 (36)	9 (25)	0.401	1 (8.3)	3 (21.4)	0.598
Other arrhytmias, n (%)	1 (2.5)	1 (3.4)	1.000	1 (8.3)	2 (14.3)	1.000	1 (4)	2 (5.6)	1.000	1 (8.3)	2 (14.3)	1.000
Valvulopathy, n (%)	9 (22.5)	0 (0)	**0.008**	2 (16.7)	2 (14.3)	1.000	7 (28)	10 (27.8)	1.000	2 (16.7)	2 (14.3)	1.000
Vasculopathy, n (%)	1 (2.5)	3 (10.3)	0.302	4 (33.3)	1 (7.1)	0.148	6 (24)	7 (19.4)	0.755	4 (33.3)	1 (7.1)	0.148
CVD (any), n (%)	28 (70)	18 (62.1)	0.606	7 (58.3)	9 (64.3)	1.000	18 (72)	26 (72.2)	1.000	7 (58.3)	9 (64.3)	1.000
Diabetes, n (%)	5 (12.5)	11 (37.9)	**0.020**	3 (25)	4 (28.6)	1.000	5 (20)	5 (13.9)	0.727	3 (25)	4 (28.6)	1.000
Ulcer, n (%)	1 (2.5)	0 (0)	1.000	0 (0)	0 (0)	-	0 (0)	1 (2.8)	1.000	0 (0)	0 (0)	-
Obesity, n (%)	2 (5.4)	0 (0)	0.502	2 (9.1)	5 (7.5)	1.000	0 (0)	6 (18.8)	**0.035**	0 (0)	1 (7.1)	1.000
Kidney disease, n (%)	11 (27.5)	1 (3.4)	**0.010**	1 (8.3)	2 (14.3)	1.000	5 (20)	5 (13.9)	0.727	1 (8.3)	2 (14.3)	1.000

Immune depression, n (%)	6 (15)	3 (10.3)	0.724	2 (16.7)	1 (7.1)	0.580	5 (20)	3 (8.3)	0.254	2 (16.7)	1 (7.1)	0.580
Liver disease, n (%)	4 (10)	1 (3.4)	0.389	1 (8.3)	0 (0)	0.462	4 (16)	4 (11.1)	0.706	1 (8.3)	0 (0)	0.462
Dementia, n (%)	6 (15)	6 (20.7)	0.542	2 (16.7)	1 (7.1)	0.580	4 (16)	5 (13.9)	1.000	2 (16.7)	1 (7.1)	0.580
Hemiplegia, n (%)	2 (5)	0 (0)	0.506	0 (0)	1 (7.1)	1.000	3 (12)	2 (5.6)	0.392	0 (0)	1 (7.1)	1.000
Psychiatric disorder, n (%)	2 (5)	3 (10.3)	0.643	2 (16.7)	2 (14.3)	1.000	0 (0)	3 (8.3)	0.262	2 (16.7)	2 (14.3)	1.000
Rheum. disease, n (%)	4 (10)	0 (0)	0.133	2 (16.7)	2 (14.3)	1.000	2 (8)	4 (11.1)	1.000	2 (16.7)	2 (14.3)	1.000
Solid tumors, n (%)	4 (10)	8 (27.6)	0.105	1 (8.3)	1 (7.1)	1.000	2 (8)	5 (13.9)	0.689	1 (8.3)	1 (7.1)	1.000
Leukemia, n (%)	1 (2.5)	4 (13.8)	0.154	1 (8.3)	0 (0)	0.462	1 (4)	1 (2.8)	1.000	1 (8.3)	0 (0)	0.462
Lymphoma, n (%)	3 (7.5)	3 (10.3)	0.690	0 (0)	0 (0)	-	1 (4)	0 (0)	0.410	0 (0)	0 (0)	-
AIDS, n (%)	2 (5)	0 (0)	0.506	0 (0)	0 (0)	-	2 (8)	0 (0)	0.164	0 (0)	0 (0)	-
COPD, n (%)	8 (20)	4 (13.8)	0.749	2 (16.7)	3 (21.4)	1.000	9 (36)	8 (22.2)	0.261	2 (16.7)	3 (21.4)	1.000
OTLT, n (%)	3 (7.5)	1 (3.4)	0.634	0 (0)	1 (7.1)	1.000	1 (4)	6 (16.7)	0.223	0 (0)	1 (7.1)	1.000
Pulmonary fibrosis, n (%)	1 (2.5)	1 (3.4)	1.000	0 (0)	0 (0)	-	1 (4)	1 (2.9)	1.000	0 (0)	0 (0)	-
Bronchiectasis, n (%)	2 (5)	1 (3.4)	1.000	0 (0)	0 (0)	-	1 (4)	0 (0)	0.417	0 (0)	0 (0)	-
Asthma, n (%)	0 (0)	2 (6.9)	0.173	1 (8.3)	3 (21.4)	0.598	3 (12)	5 (14.3)	1.000	1 (8.3)	3 (21.4)	0.598
Home treatments												
Bronchodilators, n (%)	5 (12.5)	0 (0)	0.069	2 (16.7)	4 (28.6)	0.652	7 (28)	13 (37.1)	0.581	2 (16.7)	4 (28.6)	0.652
ICS, n (%)	5 (12.5)	0 (0)	0.069	1 (8.3)	4 (28.6)	0.330	6 (24)	11 (31.4)	0.575	1 (8.3)	4 (28.6)	0.330
Immune depressants, n (%)	1 (2.6)	1 (3.4)	1.000	1 (8.3)	2 (14.3)	1.000	2 (8)	3 (8.6)	1.000	1 (8.3)	2 (14.3)	1.000
Systemic steroids, n (%)	0 (0)	1 (3.4)	0.420	0 (0)	0 (0)	-	1 (4)	3 (8.6)	0.634	0 (0)	0 (0)	-

Table S3. Anthropometrical and clinical baseline characteristics in patients with and without a coinfection depending on the viral isolate. Data are reported as median (Inter Quartile Range) if not stated otherwise. COPD = chronic Obstructive Pulmonary Disease; COVID-19 = Coronavirus 2019 Disease; ICS = inhaled corticosteroids. Statistically significant differences are highlighted in bold.
