# Supplementary material for: Burden and Risk Factors for Coinfections in Patients with a Viral Respiratory Tract Infection
Source: Pathogens. 2024 Nov 13;13(11):993. doi: 10.3390/pathogens13110993 (PMC11597400; doi:10.3390/pathogens13110993)
Supplement: Supplementary file 1 [file pathogens-13-00993-s001.zip › Supplementary Table S4_Outcomes by rivus group.pdf]

| | COVID infection, n = 69 | | | Influenza A infection (N = 101) | | | RSV infection (N = 61) | | | Other viruses infection (N = 26) | | |
|---|---|---|---|---|---|---|---|---|---|---|---|---|
| | *With coinfection (40)* | *Without coinfection (29)* | *p-value* | *With coinfection (N = 26)* | *Without coinfection (N = 75)* | *p-value* | *With coinfection (N = 25)* | *Without coinfection (N = 36)* | *p-value* | *With coinfection (N = 12)* | *Without coinfection (N = 14)* | *p-value* |
| **Imaging** | | | | | | | | | | | | |
| Chest X-ray infiltrates, n (%) | 20 (50) | 9 (33.3) | 0.214 | 12 (46.2) | 17 (23.3) | **0.043** | 9 (37.5) | 12 (34.3) | 1.000 | 3 (27.3) | 2 (16.7) | 0.640 |
| Lobar pneumonia, n (%) | 5 (12.5) | 2 (7.1) | 0.691 | 6 (23.1) | 11 (14.9) | 0.369 | 6 (25) | 4 (11.4) | 0.289 | 0 (0) | 1 (8.3) | 1.000 |
| Bilateral pneumonia, n (%) | 15 (37.5) | 10 (35.7) | 1.000 | 6 (23.1) | 3 (4.1) | **0.009** | 3 (12.5) | 6 (17.1) | 0.725 | 2 (18.2) | 1 (8.3) | 0.590 |
| Interstitial pneumonia, n (%) | 16 (40) | 11 (39.3) | 1.000 | 9 (34.6) | 9 (12.2) | **0.017** | 2 (8.3) | 6 (17.1) | 0.453 | 2 (18.2) | 1 (8.3) | 0.590 |
| Pleural effusion, n (%) | 7 (17.5) | 5 (17.9) | 1.000 | 4 (15.4) | 2 (2.7) | **0.038** | 3 (12.5) | 6 (17.1) | 0.725 | 0 (0) | 0 (0) | - |
| **Clinical presentation** | | | | | | | | | | | | |
| *Days with symptoms, n (%)* | 2 (0-7) | 6.5 (2-14) | **0.022** | 3 (2.5-7) | 2 (1-4) | **0.026** | 2.5 (0-5) | 3 (1-4) | 0.807 | 5 (2-15) | 4 (1-11) | 0.720 |
| *Fever, n (%)* | 21 (56.8) | 13 (46.4) | 0.459 | 19 (73.1) | 45 (60) | 0.345 | 11 (44) | 21 (58.3) | 0.307 | 4 (33.3) | 9 (64.3) | 0.238 |
| *Respiratory rate, bpm* | 24 (20-29) | 21 (16-24) | 0.063 | 20 (19-28) | 22 (18-27.5) | 0.959 | 20 (18-26) | 22 (10.5-38.2) | 0.144 | 20 (17.75-30.5) | 24 (20-33) | 0.429 |
| *Respiratory rate≥24, bpm* | 15 (51.7) | 6 (31.6) | 0.237 | 7 (36.8) | 20 (44.4) | 0.782 | 6 (35.3) | 9 (42.9) | 0.744 | 2 (33.3) | 3 (60.0) | 0.567 |
| *Systolic pressure, mmHg* | 130 (120-150) | 140 (130-150) | 0.323 | 125 (110-140) | 140 (125-153.75) | **0.018** | 125 (120-140) | 140 (125-160) | 0.091 | 130 (120-150) | 125 (110-143.75) | 0.608 |
| *Systolic pressure ≤130, n (%)* | 21 (53.8) | 7 (25.9) | **0.042** | 17 (65.4) | 29 (40.3) | 0.039 | 13 (56.5) | 13 (37.1) | 0.183 | 7 (63.6) | 8 (66.7) | 1.000 |
| *Systolic pressure ≤100, n (%)* | 3 (7.7) | 2 (7.4) | 1.000 | 2 (7.7) | 2 (2.8) | 0.286 | 0 (0) | 1 (2.9) | 1.000 | 1 (9.1) | 0 (0) | 0.478 |
| *Heart rate, beats/min* | 88 (78-103) | 87 (75-103) | 0.673 | 85 (78-108) | 90 (80-99) | 0.735 | 88 (72-105) | 96 (78-110) | 0.423 | 91 (72-110) | 92 (75-115) | 0.865 |
| *Heart rate ≥100, n (%)* | 11 (28.9) | 8 (28.6) | 1.000 | 8 (30.8) | 18 (24.3) | 0.605 | 7 (30.4) | 16 (44.4) | 0.412 | 4 (36.4) | 5 (38.5) | 1.000 |
| *Confusion, n (%)* | 15 (39.5) | 5 (17.9) | 0.103 | 2 (8.3) | 10 (13.7) | 0.724 | 4 (16.7) | 9 (25.0) | 0.534 | 2 (18.2) | 0 | 0.183 |
| Respiratory failure, n (%) | 24 (60) | 15 (51.7) | 0.624 | 13 (50) | 34 (45.3) | 0.820 | 15 (60) | 24 (66.7) | 0.601 | 8 (66.7) | 3 (21.4) | **0.045** |
| PaO2/FiO2, mmHg | 257 (209-290) | 280 (226-367) | 0.320 | 250 (238-300) | 276 (242-314) | 0.341 | 283 (240-321) | 261 (192-300) | 0.151 | 268 (233-307) | 318 (230-343) | 0.442 |
| Type 1 acute RF, n (%) | 17 (42.5) | 12 (41.4) | 1.000 | 12 (46.2) | 27 (36) | 0.363 | 11 (44) | 19 (52.8) | 0.605 | 6 (50) | 1 (7.1) | **0.026** |
| Type 2 acute RF, n (%) | 5 (12.5) | 3 (10.3) | 1.000 | 1 (3.8) | 8 (10.7) | 0.440 | 4 (16) | 5 (13.9) | 1.000 | 2 (16.7) | 2 (14.3) | 1.000 |
| Sodium, mmol/L | 137 (135-140) | 138 (136-140) | 0.310 | 135 (133-139) | 137 (134-139) | 0.431 | 138 (135-140) | 136 (134-139) | 0.223 | 136.5 (133-141) | 138 (136-140) | 0.437 |
| *Glycaemia, mg/dL* | 114 (87-145) | 127 (105-154) | 0.073 | 132 (104-174) | 115 (103-132) | 0.134 | 131 (96-183) | 123 (105-143) | 0.668 | 129 (110-286) | 116 (96-143) | 0.106 |

| | | | | | | | | | | | | |
|---|---|---|---|---|---|---|---|---|---|---|---|---|
| *C-reactive protein, mg/L* | 82 (39-166) | 117 (48-202) | 0.448 | 119 (38-162) | 53 (29-97) | **0.013** | 49 (22-125) | 35 (17-82) | 0.479 | 107 (26-165) | 38 (9-91) | 0.186 |
| *C-reactive protein ≤ 20, n (%)* | 3 (7.9) | 6 (20.7) | 0.160 | 4 (15.4) | 11 (15.5) | 1.000 | 5 (21.7) | 12 (33.3) | 0.391 | 2 (16.7) | 4 (30.8) | 0.645 |
| *WBCs count, cells/μL x $10^3$* | 9.585 (6.800-14.870) | 8.815 (6.572-12.272) | 0.554 | 8.955 (7.322-12.447) | 7.985 (5.690-10.445) | 0.075 | 10.095 (7.830-13.037) | 8.075 (6.912-10.605) | 0.156 | 6.615 (5.495-12.370) | 5.905 (4.540-10.195) | 0.432 |
| *WBC ≥ 8000 cell/μL, n (%)* | 26 (65.0) | 19 (67.9) | 1.000 | 17 (65.4) | 36 (50.0) | 0.251 | 17 (70.8) | 19 (52.8) | 0.189 | 5 (41.7) | 4 (28.6) | 0.683 |
| *Blood urea nitrogen, mg/dL* | 55 (36-107) | 47 (39-82) | 0.664 | 46 (37-80) | 40 (34-48) | 0.184 | 61 (36-93) | 54 (36-71) | 0.468 | 49 (26-72) | 55 (45-76) | 0.635 |
| *Creatinine, mg/dL* | 1.11 (0.82-2.05) | 1.04 (0.73-1.36) | 0.204 | 0.9 (0.71-1.12) | 0.86 (0.72-1.05) | 0.560 | 0.88 (0.69-1.48) | 0.97 (0.75-1.33) | 0.982 | 0.88 (0.82-1.16) | 0.91 (0.66-1.31) | 0.979 |
| *AST, U/L* | 20 (11-36) | 18 (10-37) | 0.848 | 20 (17-36) | 26 (17-37) | 0.622 | 17 (8-21) | 20 (13-24) | 0.090 | 18 (10-45) | 19 (12-34) | 0.928 |
| Shock, n (%) | 4 (10) | 0 (0) | 0.133 | 3 (11.5) | 1 (1.3) | 0.051 | 1 (4) | 0 (0) | 0.410 | 1 (8.3) | 0 (0) | 0.462 |
| Sepsis, n (%) | 4 (10) | 0 (0) | 0.133 | 2 (7.7) | 1 (1.3) | 0.162 | 4 (16) | 0 (0) | **0.024** | 3 (25) | 0 (0) | 0.085 |
| ***In-hospital treatments*** | | | | | | | | | | | | |
| *Antibiotics, n (%)* | 35 (87.5) | 21 (72.4) | 0.131 | 20 (80) | 46 (63.9) | 0.213 | 22 (88) | 26 (72.2) | 0.206 | 11 (100) | 9 (64.3) | **0.046** |
| *Systemic steroids, n (%)* | 27 (67.5) | 16 (55.2) | 0.325 | 13 (52) | 36 (50) | 1.000 | 17 (68) | 23 (63.9) | 0.790 | 9 (81.8) | 7 (53.8) | 0.211 |
| *Inhaled steroids, n (%)* | 13 (32.5) | 4 (13.8) | 0.094 | 12 (48) | 42 (58.3) | 0.484 | 16 (64) | 26 (74.3) | 0.409 | 5 (45.5) | 10 (71.4) | 0.241 |
| *Oseltamivir, n (%)* | 0 | 0 | - | 17 (65.4) | 45 (60) | 0.816 | 3 (12) | 5 (13.9) | 1.000 | 0 (0) | 2 (14.3) | 0.483 |
| *Antivirals (other), n (%)* | 21 (52.5) | 14 (48.3) | 0.809 | 3 (11.5) | 0 (0) | **0.017** | 4 (16) | 1 (2.8) | 0.149 | 2 (16.7) | 1 (7.1) | 0.580 |
| ***Respiratory support*** | | | | | | | | | | | | |
| Oxygen therapy, n (%) | 34 (85) | 18 (62.1) | **0.046** | 23 (88.5) | 50 (66.7) | **0.042** | 18 (72) | 29 (80.6) | 0.540 | 10 (83.3) | 6 (42.9) | 0.051 |
| HFNC, n (%) | 4 (10) | 2 (6.9) | 1.000 | 6 (23.1) | 1 (1.3) | **0.001** | 4 (16) | 2 (5.6) | 0.216 | 3 (25) | 0 (0) | 0.085 |
| CPAP, n (%) | 9 (22.5) | 5 (17.2) | 0.764 | 4 (15.4) | 1 (1.3) | **0.015** | 3 (12) | 3 (8.3) | 0.682 | 1 (8.3) | 2 (14.3) | 1.000 |
| NIV, n (%) | 2 (5) | 1 (3.4) | 1.000 | 5 (19.2) | 3 (4) | **0.025** | 4 (16) | 6 (16.7) | 1.000 | 3 (25) | 0 (0) | 0.085 |
| ETI, n (%) | 6 (15) | 2 (6.9) | 0.453 | 5 (19.2) | 2 (2.7) | **0.012** | 3 (12) | 1 (2.8) | 0.296 | 3 (25) | 0 (0) | 0.085 |
| ***Outcome*** | | | | | | | | | | | | |
| *Length of stay, days* | 22 (15-30) | 10 (3-16) | **<0.001** | 26 (11-32) | 5 (1-9) | **<0.001** | 19 (10-38) | 8 (4-16) | **<0.001** | 25 (23-53) | 8 (2-18) | **0.001** |
| Time to coinfection, days | 5 (2-10) | - | - | 1 (0-5) | - | - | 2 (0-5) | - | - | 3.5 (1.0-6.2) | 0 | - |
| *Severe disease, n (%)* | 35 (87.5) | 19 (65.5) | **0.040** | 23 (88.5) | 50 (66.7) | **0.042** | 23 (92) | 29 (80.6) | 0.286 | 10 (83.3) | 6 (42.9) | 0.051 |
| Time to ICU admission, days | 2 (0-3) | 1 (0-1) | 0.383 | 4.5 (1-7) | - | 0.222 | 1 (0-1) | - | 0.500 | 4.0 (1.0-4.0) | 0 | - |
| *ICU, n (%)* | 7 (17.5) | 3 (10.3) | 0.502 | 8 (30.8) | 2 (2.7) | **<0.001** | 3 (12) | 1 (2.8) | 0.296 | 3 (25) | 0 (0) | 0.085 |

| | | | | | | | | | | | | |
|---|---|---|---|---|---|---|---|---|---|---|---|---|
| *Tracheostomy, n (%)* | 3 (7.5) | 0 (0) | 0.258 | 3 (11.5) | 1 (1.3) | 0.051 | 3 (12) | 0 (0) | 0.064 | 2 (16.7) | 0 (0) | 0.203 |
| *In-hospital death, n (%)* | 7 (17.5) | 3 (10.3) | 0.502 | 5 (19.2) | 1 (1.3) | **0.004** | 3 (12) | 1 (2.8) | 0.296 | 2 (16.7) | 0 (0) | 0.203 |
| *ICU death, n (%)* | 7 (17.5) | 3 (10.3) | 0.502 | 5 (19.2) | 1 (1.3) | **0.004** | 3 (12) | 1 (2.8) | 0.296 | 2 (16.7) | 0 (0) | 0.203 |

**Table S4.** Characteristics at emergency department presentation, in-hospital treatments and clinical outcomes in patients with and without a coinfection depending on the viral isolate. Patients with a positive viral swab and a coinfection were compared with the rest of the tested patients (column C). Data are reported as median (Inter Quartile Range) if not stated otherwise. aRF = acute respiratory failure; CRP = C reactive protein; ICU = Intensive Care Unit; ETI = Endotracheal Intubation; NIV = non invasive ventilation; CPAP = continuous positive airway pressure; HFNC = high flow nasal cannula; ICS = inhaled corticosteroids; FiO2 = fractional inhaled oxygen; PaO2 = arterial partial pressure of oxygen; SBP = systolic blood pressure; WBC = white blood cells count. Statistically significant differences are highlighted in bold.
