# Supplementary material for: Burden and Risk Factors for Coinfections in Patients with a Viral Respiratory Tract Infection
Source: Pathogens. 2024 Nov 13;13(11):993. doi: 10.3390/pathogens13110993 (PMC11597400; doi:10.3390/pathogens13110993)
Supplement: Supplementary file 1 [file pathogens-13-00993-s001.zip › Supplementary Table S5_Outcomes in MDR coinfection.pdf]

| | Infection/Coinfection with MDR bacteria | | |
|---|---|---|---|
| Outcome | Viral infection YES (N = 34) | Viral infection NO (N = 47) | p-value |
| Severe disease, n (%) | 32 (94.1) | 38 (80.9) | 0.079 |
| Sepsis, n (%) | 2 (5.9) | 13 (27.7) | **0.011** |
| Shock, n (%) | 2 (5.9) | 6 (12.8) | 0.264 |
| Tracheostomy, n (%) | 3 (8.8) | 0 (0) | 0.070 |
| aRF, n (%) | 20 (58.8) | 26 (56.5) | 0.466 |
| Oxygen, n (%) | 31 (91.2) | 36 (76.6) | 0.076 |
| HFNC, n (%) | 5 (14.9) | 7 (14.7) | 0.619 |
| CPAP, n (%) | 5 (14.7) | 1 (2.1) | **0.044** |
| NIV, n (%) | 4 (11.8) | 3 (6.4) | 0.322 |
| ETI, n (%) | 6 (17.6) | 3 (6.4) | 0.109 |
| ICU, n (%) | 7 (20.6) | 4 (8.5) | 0.109 |
| Death ICU, n (%) | 9 (26.5) | 4 (8.5) | **0.031** |
| Death in-hospital, n (%) | 9 (26.5) | 4 (8.5) | **0.031** |

**Table S5.** Clinical outcomes in patients with a MDR infection in case of underlying viral infection (N = 29) compared with patients without a viral positive swab (N = 45). aRF = acute respiratory failure; ETI = Endotracheal Intubation; ICU = Intensive Care Unit; MDR = multi drug resistant (it includes vancomycin resistant *S. aureus* and carbapenem resistant gram negative bacteria); NIV = non invasive ventilation; CPAP = continuous positive airway pressure; HFNC = high flow nasal cannula. Statistically significant differences are highlighted in bold.
