# Supplementary figures and images for: Burden and Risk Factors for Coinfections in Patients with a Viral Respiratory Tract Infection
Source: Pathogens. 2024 Nov 13;13(11):993. doi: 10.3390/pathogens13110993 (PMC11597400; doi:10.3390/pathogens13110993)

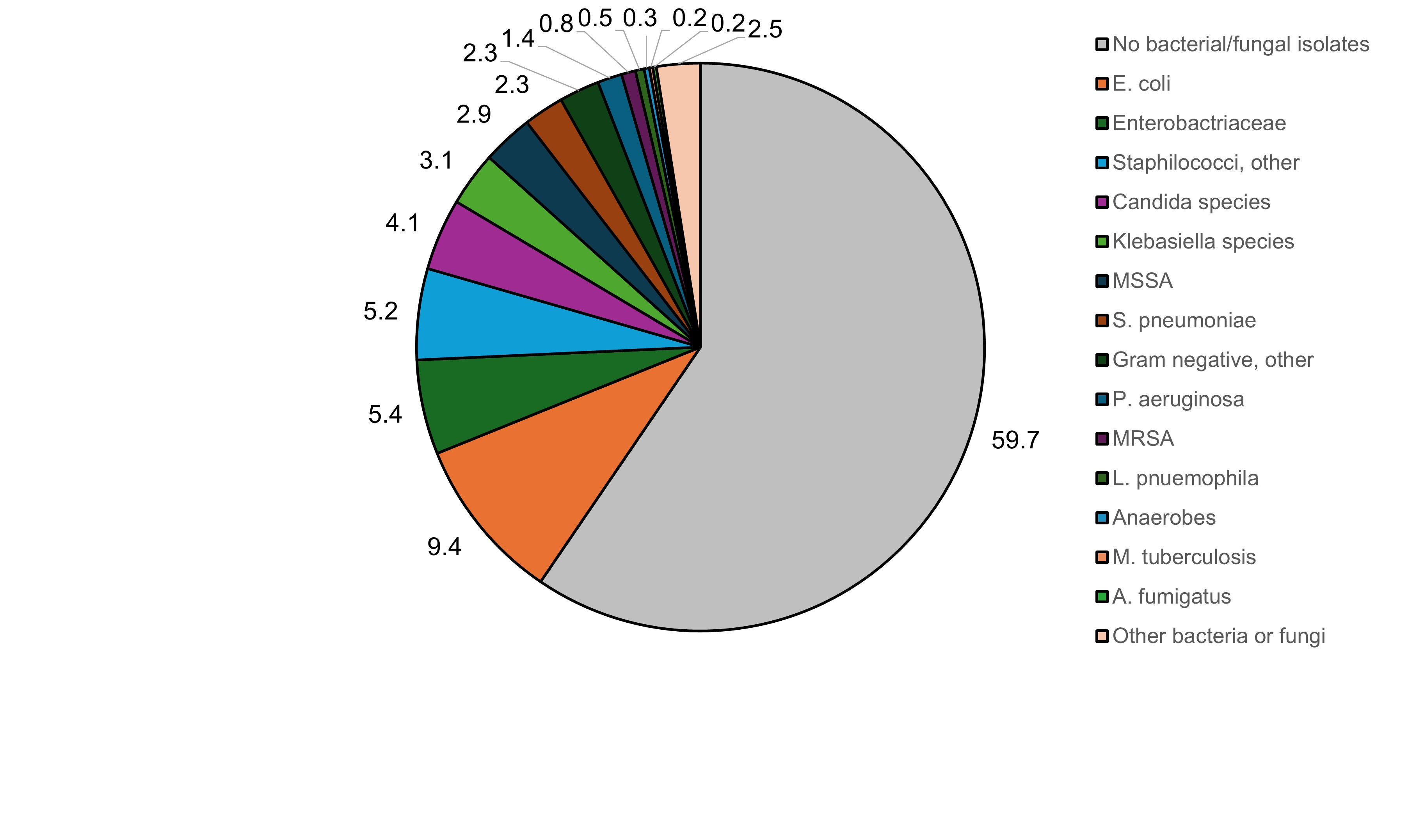

Supplement: Supplementary file 1 [file pathogens-13-00993-s001.zip › Supplementary Figure S1.jpg]

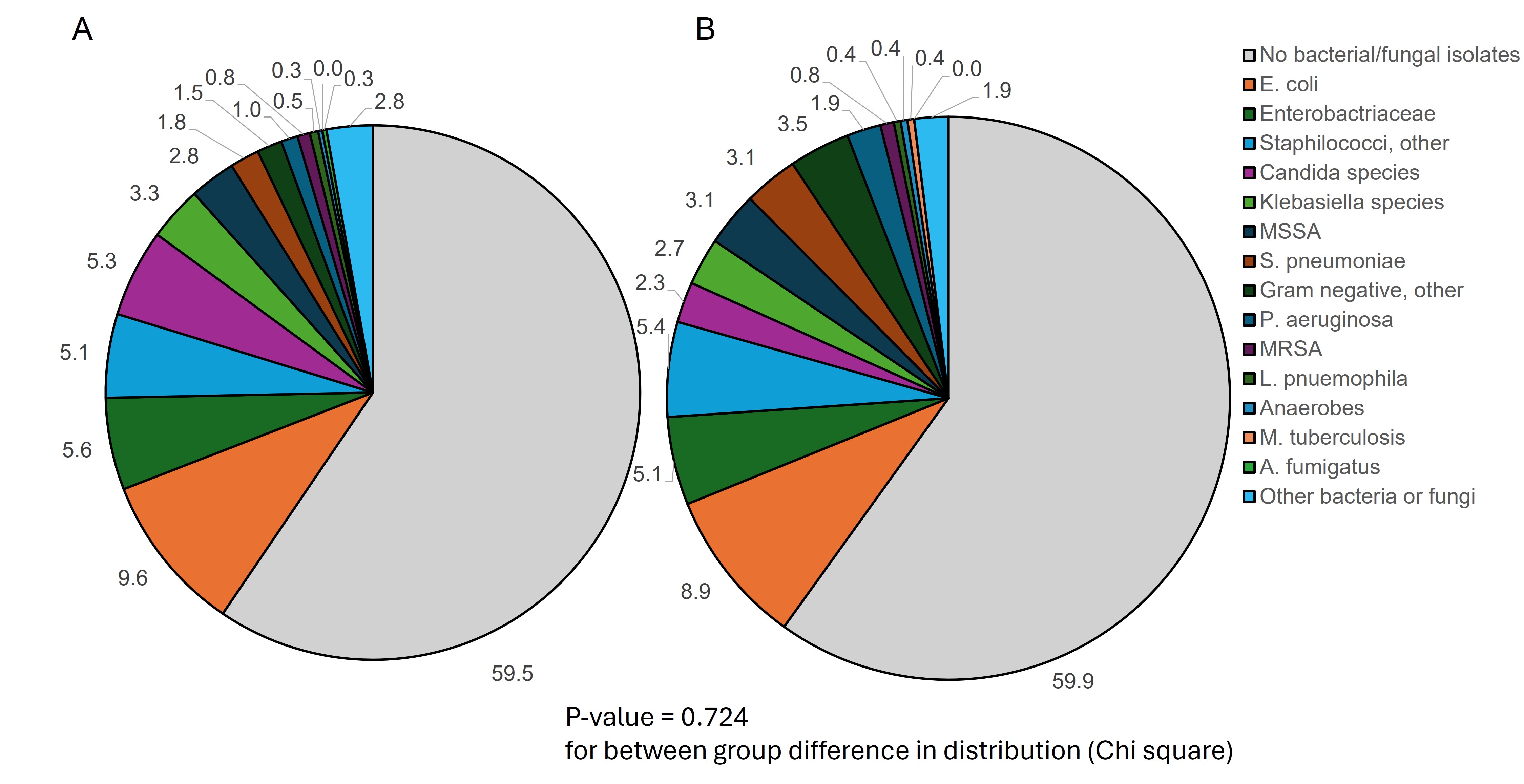

Supplement: Supplementary file 1 [file pathogens-13-00993-s001.zip › Supplementary Figure S2.jpg]

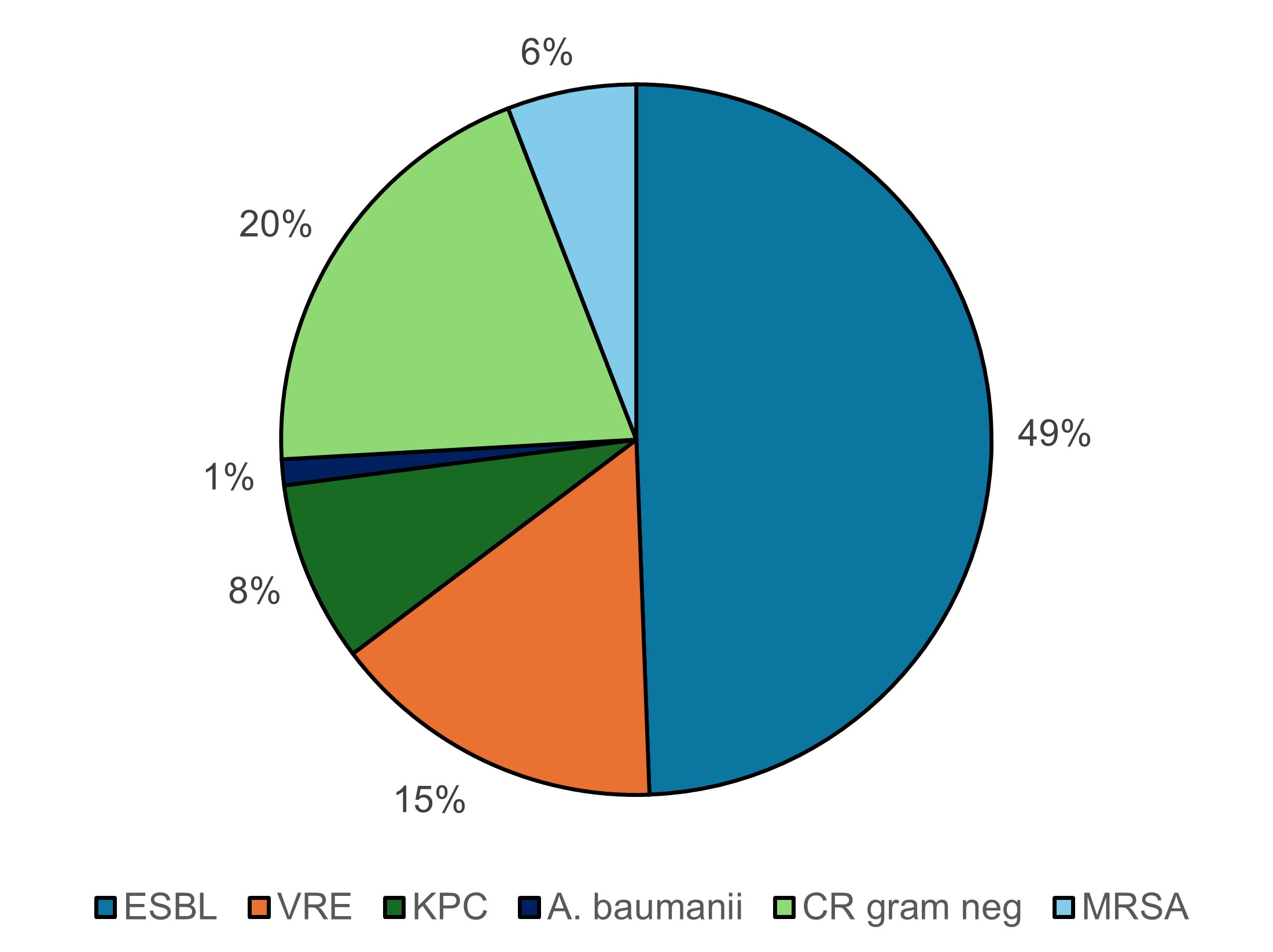

Supplement: Supplementary file 1 [file pathogens-13-00993-s001.zip › Supplementary Figure S3.jpg]

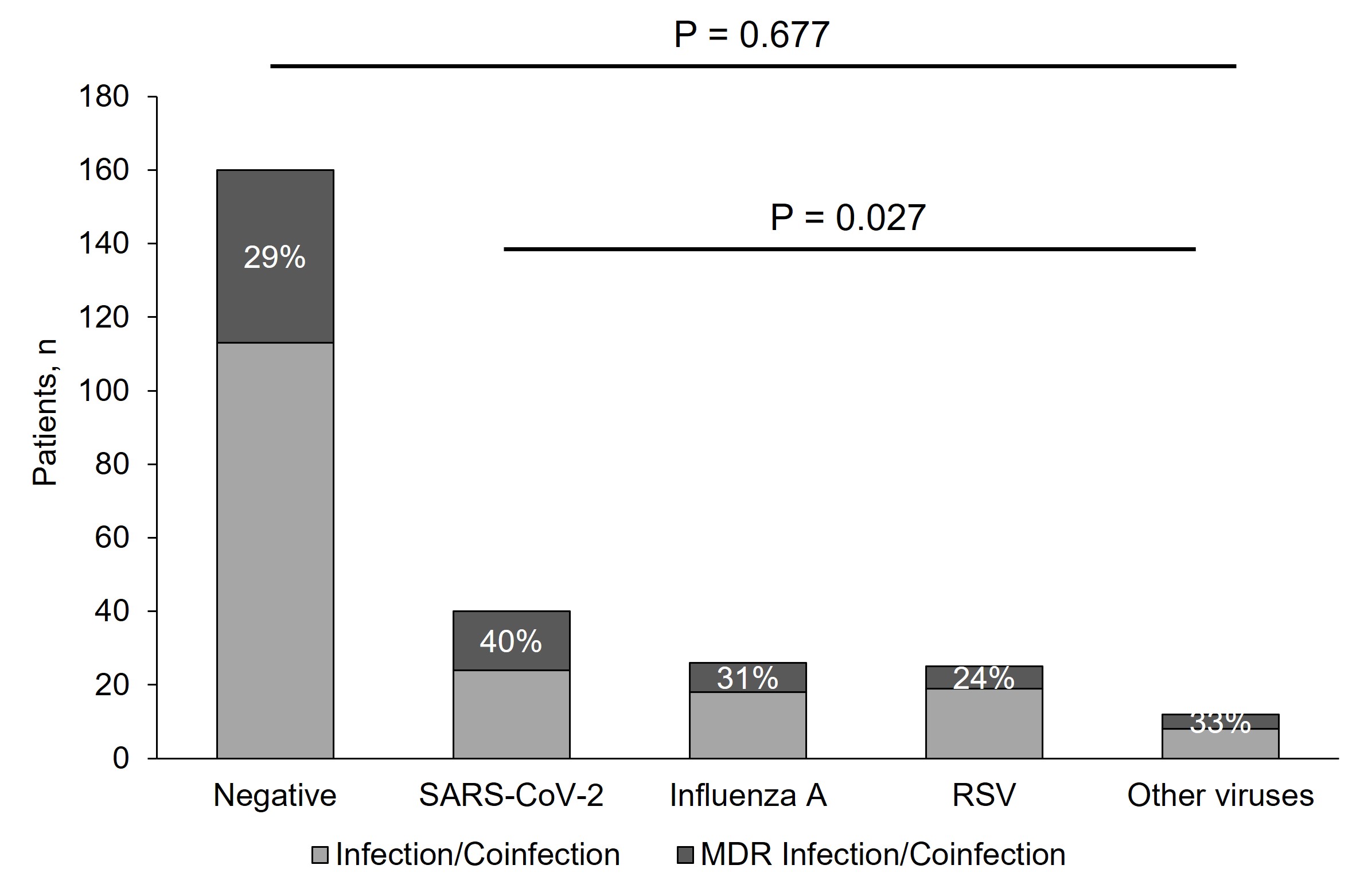

Supplement: Supplementary file 1 [file pathogens-13-00993-s001.zip › Supplementary Figure S4.jpg]

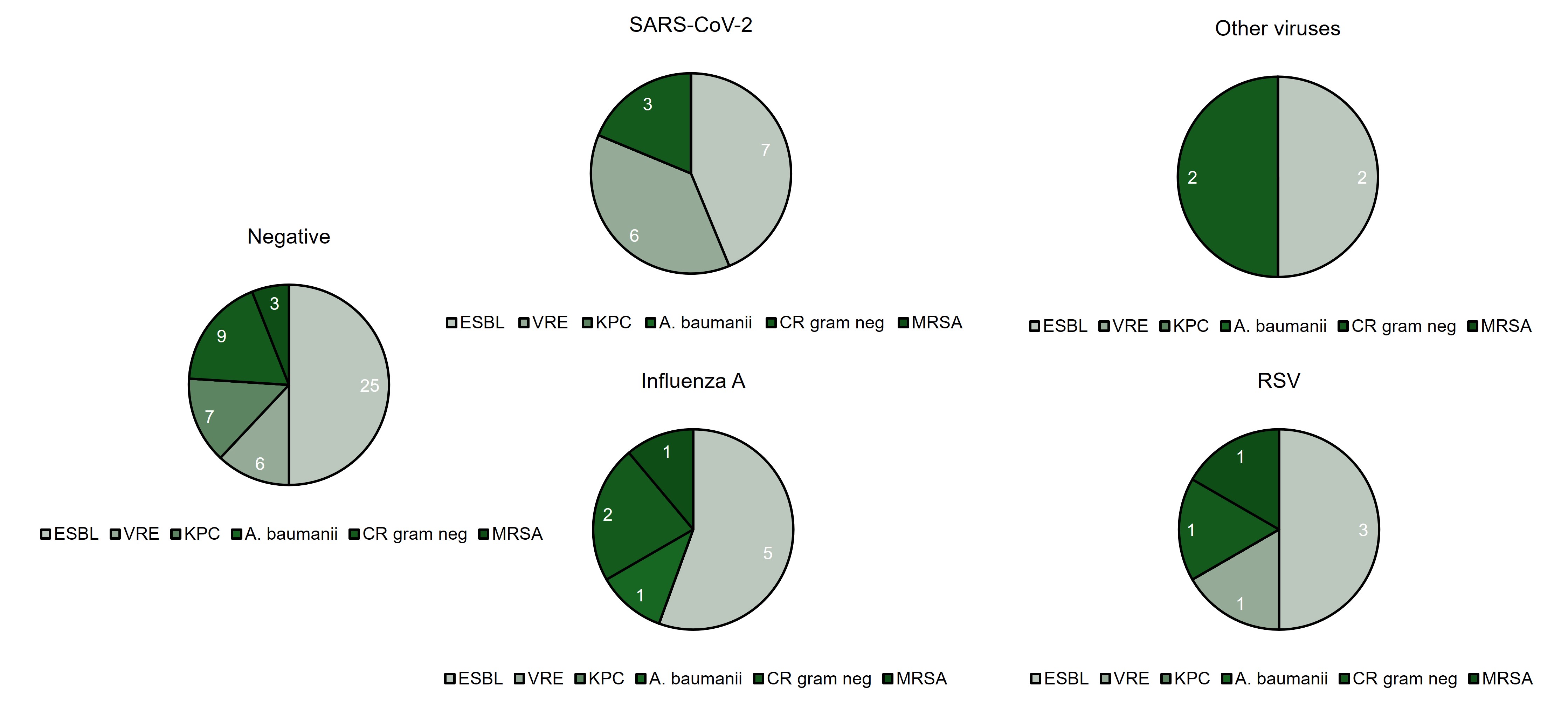

Supplement: Supplementary file 1 [file pathogens-13-00993-s001.zip › Supplementary Figure S5.jpg]
